# Genetic polymorphisms in innate immunity genes influence predisposition to tick-borne encephalitis

**DOI:** 10.1007/s13365-023-01182-8

**Published:** 2023-10-28

**Authors:** Andrea Fortova, Andrey V. Barkhash, Martina Pychova, Lenka Krbkova, Martin Palus, Jiri Salat, Daniel Ruzek

**Affiliations:** 1 Department of Infectious Diseases and Preventive Medicine, Veterinary Research Institute, CZ-62100 Brno, Czechia; 2 Department of Experimental Biology, Faculty of Science, Masaryk University, CZ-62500 Brno, Czechia; 3 Federal Research Center Institute of Cytology and Genetics, Siberian Branch, Russian Academy of Sciences, 10 Lavrentyeva Ave, Novosibirsk, 630090 Russia; 4 Department of Infectious Diseases, University Hospital Brno and Faculty of Medicine, Masaryk University, CZ-62500 Brno, Czechia; 5 Department of Children’s Infectious Disease, Faculty of Medicine and University Hospital, Masaryk University, CZ-61300 Brno, Czechia; 6 Institute of Parasitology, Biology Centre of the Czech Academy of Sciences, CZ-37005 Ceske Budejovice, Czechia; 7 Joint Faculty of Veterinary Medicine, Yamaguchi University, Yamaguchi City, Japan

**Keywords:** Tick-borne encephalitis, Genetics, Immunity genes, Single-nucleotide polymorphism, Predisposition

## Abstract

Tick-borne encephalitis (TBE) is a neuroviral disease that ranges in severity from a mild febrile illness to a severe and life-threatening meningoencephalitis or encephalomyelitis. There is increasing evidence that susceptibility to tick-borne encephalitis virus (TBEV)-induced disease and its severity are largely influenced by host genetic factors, in addition to other virus- and host-related factors. In this study, we investigated the contribution of selected single nucleotide polymorphisms (SNPs) in innate immunity genes to predisposition to TBE in humans. More specifically, we investigated a possible association between SNPs rs304478 and rs303212 in the gene Interferon Induced Protein With Tetratricopeptide Repeats 1 (*IFIT1*), rs7070001 and rs4934470 in the gene Interferon Induced Protein With Tetratricopeptide Repeats 2 (*IFIT2*), and RIG-I (Retinoic acid-inducible gene I) encoding gene *DDX58* rs311795343, rs10813831, rs17217280 and rs3739674 SNPs with predisposition to TBE in population of the Czech Republic, where TBEV is highly endemic. Genotypic and allelic frequencies for these SNPs were analyzed in 247 nonimmunized TBE patients and compared with 204 control subjects. The analysis showed an association of *IFIT1* rs304478 SNP and *DDX58* rs3739674 and rs17217280 SNPs with predisposition to TBE in the Czech population indicating novel risk factors for clinical TBE but not for disease severity. These results also highlight the role of innate immunity genes in TBE pathogenesis.

## Introduction

Tick-borne encephalitis (TBE), a neuroviral disease transmitted by ticks, is common in much of the forested areas of Europe and Asia (Mansfield et al. 2009; Ruzek et al. 2019). More than 10,000 clinical cases of TBE are reported each year, although effective vaccines against TBE are available (Mansfield et al. 2009; Ruzek et al. 2019). TBE is caused by the tick-borne encephalitis virus (TBEV; *Orthoflavivirus encephalitidis*), which belongs to the Flaviviridae family and the *Orthoflavivirus* genus (Zerbini et al. 2023). The clinical presentation of TBE varies from a mild febrile illness to severe and life-threatening meningoencephalitis or encephalomyelitis (Bogovic and Strle 2015). In addition, a significant proportion of TBE infections are thought to be asymptomatic (Bojkiewicz et al. 2022; Gritsun et al. 2003). However, what determines the predisposition to TBEV-mediated disease and the severity of TBE remains poorly understood, but it is thought that both viral and host factors play important roles. Viral factors include the virulence hypothesis, which states that some TBEV strains are more virulent than others and lead to more severe disease (Leonova et al. 2013, 2014). Age and immune status are among the most important host factors determining the severity of TBE, as older individuals are more susceptible to more severe forms of TBE with higher mortality rates than younger individuals (Jelenik et al. 2010). There is increasing evidence that host genetic factors also play a critical role in determining predisposition to clinically manifest TBE and to varying degrees of severity of infection (Barkhash et al. 2016, 2018a, b; 2012, 2010, 2013; Czupryna et al. 2017; Kindberg et al. 2008; Mickienė et al. 2014).

Animal models of TBE have identified several candidate genes that influence survival after TBEV infection, including *Oas1b*, *Cd33*, *Klk1b22*, *Siglece*, *Klk1b16*, *Fut2*, *Grwd1*, *Abcc6*, *Otog*, and *Mkrn3* (Palus et al. 2018, 2013). The predisposition to TBE in humans has been studied in detail, especially in the Russian population. Specifically, candidate gene analyses demonstrated that single nucleotide polymorphisms (SNPs) in the *OAS2*, *OAS3*, *CD209*, *TLR3*, *IL28B*, *MMP9*, and *IL10* genes, which encode key components of the innate immune response, are associated with predisposition to TBE in Russian residents from Novosibirsk and Irkutsk (Barkhash et al. 2016, 2018a, b; 2012, 2010, 2013). Also, a functional Toll-like receptor 3 gene (*TLR3*) and nonfunctional *CCR5Δ32* mutation were found to be risk factors for TBE (Kindberg et al. 2008; Mickienė et al. 2014). However, our recent work shows that genetic determinants may differ greatly between different populations, as the role of the *MMP9* SNP in TBE predisposition clearly demonstrated in Russian residents was not observed in Czech TBE patients (Fortova et al. 2023). This highlights the importance of studying genetic markers of TBE predisposition in different populations living in TBE endemic areas.

The Czech Republic (population 10.5 million) is among the countries with the highest TBE incidence in Europe outside Russia. Since the early 1990s, there has been a steep increase in reported TBE cases in the Czech Republic, and this situation continues to this day, with some annual fluctuations (Kriz et al. 2012). The previous peak of cases reported per year was 1029 cases in 2006, and in the last 10 years the number of cases has fluctuated between 355 (2015) and 855 (2020) with a median of 628 cases per year. The genetic factors determining the predisposition to TBE in the Czech population are largely unknown. The aim of this study was to investigate a possible association between polymorphisms in key innate immunity genes and susceptibility to TBEV-mediated diseases in the Czech population.

Interferon (IFN) secretion from virus-infected cells is a key feature of the host’s antiviral immune response (Zhou et al. 2013). In TBE patients, several IFNs were found to be up-regulated. Notably, there was a trend toward higher expression of IFNβ and IFNλ3 in patients with a milder clinical presentation of the disease (Grygorczuk et al. 2015). Conversely, the highest levels of IFNγ in the cerebrospinal fluid were observed in patients with severe TBE presenting with disorders of consciousness (Kondrusik et al. 2005). IFNs exert their antiviral effects through inducing antiviral proteins. Hundreds of IFN-stimulated genes include the *IFITs* (IFN-induced protein with tetratricopeptide repeats) family (Zhou et al. 2013).

Here, we examined selected SNPs in the genes *IFIT1*, *IFIT2*, and RIG-I (Retinoic acid-inducible gene I) encoding gene *DDX58*. The *IFIT1* and *IFIT2* genes encode IFIT1 and IFIT2, respectively, and both are located on chromosome 10 (Zhou et al. 2013). The IFIT1 protein specifically binds single-stranded RNA carrying a 5' triphosphate group, acting as a sensor for viral single-stranded RNAs and inhibiting the expression of viral messenger RNAs. The IFIT2 protein inhibits the expression of viral messenger RNAs lacking 2'-O-methylation of the 5'-cap and may promote apoptosis (Zhou et al. 2013). The Retinoic Acid-inducible Gene I (RIG-I) protein, a member of RIG-I-like receptor family, is encoded by *DDX58* gene and is involved in the recognition of viral double-stranded RNA and in the regulation of the antiviral innate immune response (Brisse and Ly 2019).

Because the severity and outcome of TBEV infection depend particularly on the efficiency of innate and adaptive immunity in suppressing viral replication, we hypothesized that polymorphisms in the human *IFIT1*, *IFIT2*, and *DDX58* genes might have major effects on the outcome of TBEV infection.

## Patients and methods

### Patients and blood sampling

Blood samples were obtained from 247 patients (aged 3–87 years, mean 47.98 years), who were divided into two subgroups: Patients with meningitis (TBE-mening; *n* = 129, 64 male, aged 3–87 years, mean 44.46 years) and patients with more severe forms of TBE (encephalitis, meningoencephalitis, encephalomyelitis; TBE-enc; *n* = 118, 69 male, aged 18–84 years, mean 51.51 years). TBE diagnosis was made according to the case definition and based on the following criteria: (i) the presence of clinical signs of meningitis, meningoencephalitis, or meningoencephalomyelitis and epidemiologic association; (ii) CSF pleocytosis (> 5 cells/µl); and (iii) the presence of TBEV-specific IgM and IgG antibodies in serum or TBE-specific IgM antibodies in CSF. None of the patients had been vaccinated against TBE. Blood samples from 204 blood donors (161 male) living in the same area as the patients served as negative controls. Males and females in control group were distinguished based on sex determining region Y (SRY) located in the short arm of human chromosome Y using gb Genetic Sry kit (Generi Biotech, Hradec Kralove, Czech Republic). Total amount of genomic DNA in reaction mixture was in a range of 4–400 ng. The protocol of qPCR was set up according to manufacturer’s instructions. All patients and blood donors lived in the South Moravia region, Czech Republic.

### SNP genotyping

Genomic DNA was extracted from whole blood using the QIAamp DNA Blood Mini Kit (Qiagen, Hilden, Germany) according to the manufacturer’s instructions. The TaqMan® SNP Genotyping Assay (ThermoFisher Scientific, Waltham, MA, USA) was used to genotype SNPs rs304478, rs303212 polymorphism in the *IFIT1* gene, rs7070001, rs4934470 polymorphism in the *IFIT2* gene, and rs11795343, rs10813831, rs17217280, and rs3739674 polymorphism in the *DDX58* gene (Table 1). Amplification mixes contained 10 ng of DNA, 1xTaqMan® Genotyping Master Mix (ThermoFisher Scientific, Waltham, MA, USA), 1xTaqMan® SNP Genotyping Assay (ThermoFisher Scientific, Waltham, MA, USA) including allele-specific probe and primers labelled with the two allele-specific fluorescent reporter dyes VIC and FAM, and Milli-Q water in a 10 µl reaction. PCR reactions were performed using the LightCycler® 480 System (Roche, Hague Rd., Indianapolis, USA) in 96-well plates. The PCR profile was as follows: 10 min at 95 °C and 40 cycles of 15 s at 95 °C followed by 60 s at 60 °C. There were two negative controls on each plate. Post-read allele discrimination analysis was performed after amplification.

**Table 1:** Genotyping assays for studied polymorphisms

**Gene**	**Gene name**	**SNP**	**Assay_ID**	**Nucleotide change**
*IFIT1*	Interferon induced protein with tetratricopeptide repeats 1	rs304478	C___2725110_10	G/T
*IFIT1*	Interferon induced protein with tetratricopeptide repeats 1	rs303212	C___2329115_20	C/T
*IFIT2*	Interferon induced protein with tetratricopeptide repeats 2	rs7070001	C___2734224_20	C/T
*IFIT2*	Interferon induced protein with tetratricopeptide repeats 2	rs4934470	C___2734217_10	C/T
*DDX58*	DEXD/H-box helicase 58	rs11795343	C__31933389_20	C/T
*DDX58*	DEXD/H-box helicase 58	rs10813831	C___1552406_10	A/G
*DDX58*	DEXD/H-box helicase 58	rs17217280	C__25963266_10	A/T
*DDX58*	DEXD/H-box helicase 58	rs3739674	C__27471598_10	C/G

### Statistical analyses

Genotypic and allelic SNP frequencies were compared between the studied TBE patient and control groups with the χ2 test using GraphPad Prism 9 version 9.3.0 (GraphPad Software, La Jolla, CA, USA). The difference between two groups was considered significant if the *p*-value was less than 0.05.

## Results and discussion

IFNs play a key role in restriction of TBEV infection, but their antiviral activities are exerted via activation of antiviral proteins rather than directly. Among hundreds of IFN-stimulated genes, IFITs play a central role in the antiviral response. The human *IFIT* gene family generally consists of four members (*IFIT1*, *IFIT2*, *IFIT3*, and *IFIT5*) (Zhou et al. 2013). IFIT1 and IFIT2 are among the proteins that are readily activated after TBEV infection (Selinger et al. 2022). Pattern recognition receptors such as RIG-I, which in humans is encoded by the gene *DDX58*, play a key role in IFN production following TBEV infection (Miorin et al. 2012). Therefore, we focused our attention on these major players in the innate immune response to TBEV infection and performed an analysis of genotypic and allelic frequencies of polymorphisms in selected SNPs in the human *IFIT1*, *IFIT2*, and *DDX58* genes that may have major implications for the outcome of TBEV infection.

The genotypic and allelic frequencies of SNPs rs304478 and rs303212 for the *IFIT1* gene, rs7070001 and rs4934470 for the *IFIT2* gene, and rs311795343, rs10813831, rs17217280, and rs3739674 for the *DDX58* gene in TBE patients are shown in Table 2. The group of TBE patients included the subgroups of TBE-mening and TBE-enc. Frequencies were compared between the total number of TBE patients and the control group, and between TBE-enc patients and either TBE-mening patients or the control group.

**Table 2:** Genotyping and allelic frequencies for the *IFIT1* gene rs304478 and rs303212, *IFIT2* gene rs7070001, rs4934470, and *DDX58* gene rs11795343, rs10813831, rs17217280, and rs3739674 single nucleotide polymorphisms (SNPs) in tick-borne encephalitis (TBE) patients with different clinical manifestation and in the control group

**Genes, SNP**	**Control group**^**a**^	**TBE patients**^**a**^			***P*** **values**	
		**All**	**TBE-mening**	**TBE-enc**	**(TBE all/control)**	**(TBE-enc/control)**
***IFIT1*, rs304478**						
TT	23.5 (48)	28.3 (70)	26.4 (34)	30.5 (36)	n.s	n.s
GG	28.9 (59)	49.4 (122)	51.1 (66)	47.5 (56)	< 0.001	0.001
GT	47.6 (97)	22.3 (55)	22.5 (29)	22.0 (26)	< 0.001	< 0.001
T	47.3	39.5	37.6	41.5	0.018	n.s
G	52.7	60.5	62.4	58.5	0.018	n.s
N^b^	204	247	129	118	n.s	n.s
***IFIT1*, rs303212**						
TT	56.8 (116)	54.3 (134)	59.7 (77)	48.3 (57)	n.s	n.s
CC	16.2 (33)	16.6 (41)	14.7 (19)	18.6 (22)	n.s	n.s
TC	27.0 (55)	29.1 (72)	25.6 (33)	33.1 (39)	n.s	n.s
T	70.3	68.8	72.5	64,8	n.s	n.s
C	29.7	31.2	27.5	35.2	n.s	n.s
N	204	247	129	118	n.s	n.s
***IFIT2*, rs7070001**						
TT	7.4 (15)	7.7 (19)	5.4 (7)	10.2 (12)	n.s	n.s
CC	58.8 (120)	55.5 (137)	58.1 (75)	52.5 (62)	n.s	n.s
TC	33.8 (69)	36.8 (91)	36.4 (47)	37.3 (44)	n.s	n.s
T	24.3	26.1	23.6	28.8	n.s	n.s
C	75.7	73.9	76.4	71.2	n.s	n.s
N	204	247	129	118	n.s	n.s
***IFIT2*, rs4934470**						
TT	1.4 (3)	2.8 (7)	2.3 (3)	3.4 (4)	n.s	n.s
CC	61.8 (126)	68.0 (168)	69.0 (89)	66.9 (79)	n.s	n.s
TC	36.8 (75)	29.2 (72)	28.7 (37)	29.7 (35)	n.s	n.s
T	19.8	17.4	16.7	18.2	n.s	n.s
C	80.2	82.6	83.3	81.8	n.s	n.s
N	204	247	129	118	n.s	n.s
***DDX58*, rs11795343**						
TT	41.7 (85)	36.4 (90)	34.1 (44)	39.0 (46)	n.s	n.s
CC	15.7 (32)	22.7 (56)	20.9 (27)	24.6 (29)	n.s	n.s
CT	42.6 (87)	40.9 (101)	45.0 (58)	36.4 (43)	n.s	n.s
T	63.0	56.9	56.6	57.2	n.s	n.s
C	37.0	43.1	43.4	42.8	n.s	n.s
N	204	247	129	118	n.s	n.s
***DDX58*, rs10813831**						
GG	0 (0)	1.2 (3)	0.8 (1)	1.7 (2)	n.s	n.s
AA	31.4 (64)	28.6 (70)	23.4 (30)	34.2 (40)	n.s	n.s
GA	68.6 (140)	70.2 (172)	75.8 (97)	64.1 (75)	n.s	n.s
G	34.3	36.3	38.7	33.8	n.s	n.s
A	65.7	69.7	61.3	66.2	n.s	n.s
N	204	245	128	117	n.s	n.s
***DDX58*, rs17217280**						
TT	4.4 (9)	0 (0)	0 (0)	0 (0)	0.001	0.021
AA	77.0 (157)	72.9 (180)	71.3 (92)	74.6 (88)	n.s	n.s
TA	18.6 (38)	27.1 (67)	28.7 (37)	25.4 (30)	0.034	n.s
T	13.7	13.6	14.3	12.7	n.s	n.s
A	86.3	86.4	85.7	87.3	n.s	n.s
N	204	247	129	118	n.s	n.s
***DDX58*, rs3739674**						
GG	14.7 (30)	13.8 (34)	12.4 (16)	15.3 (18)	n.s	n.s
CC	55.4 (113)	71.6 (177)	72.1 (93)	71.2 (84)	< 0.001	0.005
GC	29.9 (61)	14.6 (36)	15.5 (20)	13.5 (16)	< 0.001	0.001
G	29.7	21.1	20.2	22	n.s	n.s
C	70.3	78.9	79.8	78	0.003	0.035
N	204	247	129	118	0.003	0.035

^a^Number of subjects with a given genotype (Genotype (allele) frequency, % (number))

^b^N, number of individuals

*n.s.* not significant

For the *IFIT1* rs304478 SNP, significant differences in the frequency of G/G homozygotes were found between all TBE patients (28.9%) and the control group (49.4%) (*P* < 0.001). A significant difference (*P* = 0.001) was found between the TBE-enc patients (47.5%) and the control group. In addition, the frequency of G/T heterozygotes was significantly higher in the control group (47.6%) than in TBE patients (22.3%) or in TBE-enc patients (22.0%) (*P* < 0.001). In addition, the frequency of the G allele for this SNP is significantly lower in the control group (52.7%) compared with all TBE patients (60.5%) (*P* = 0.018). Similarly, the rs304478 SNP has been found previously to be associated with a sustained virological response in hepatitis C infection (Lopez-Rodriguez et al. 2011). No statistically significant differences, however, were observed in genotype and/or allele frequencies in *IFIT1* rs303212 SNP.

For *IFIT2*, no statistically significant differences were found between the compared groups in genotype or allele frequencies for SNPs rs707001 and rs4934470.

For the *DDX58* gene SNP rs3739674, in TBE patients (71.6%), including TBE-enc (71.24%), a significant increase in the frequency of C/C homozygotes was found compared with the control group (55.4%) (*P* < 0.001 and 0.005). For the same SNP, the frequency of G/C heterozygotes is significantly lower in TBE patients (14.6%), especially in TBE-enc patients (13.5%) compared with the control group (29.9%) (*P* < 0.001 and *P* = 0.001, respectively). In addition, a significant increase in C allele frequency for this SNP was detected in TBE patients (78.9%) and TBE-enc patients (78.0%) compared with the control group (70.3%) (*P* = 0.003 and 0.035, respectively). Previous studies reported an association of *DDX58* rs3739674 polymorphism with an increased risk of enterovirus 71-associated hand, foot, and mouth disease in Chinese children and with the disease severity (Li et al. 2018, 2022).

For the *DDX58* gene SNP rs17217280, a significant increase in T/T homozygotes was observed only in the control group (4.4%) compared with all TBE patients (0%) (*P* = 0.001) and with TBE-enc patients (*P* = 0.021). For the same SNP, significant differences in the frequencies of T/A heterozygotes were observed between TBE patients (27.1%) and the control group (18.6%) (*P* = 0.034).

Taken together, this study found that SNPs in the *IFIT1* and *DDX58* genes are associated with predisposition to TBE in the Czech population representing new risk factors for TBE. Our results provide further evidence for the importance of host genetic factors in predisposition to TBE and add new candidate genes to the list of previously identified genetic polymorphisms that may be involved in this process in humans. Our results also suggest that genes associated with innate immune response may have a major impact on overall susceptibility to clinically manifest TBEV infection. The significance of IFIT1, IFIT2, and RIG-I in flavivirus infections has been well-documented (Guo et al. 2018; Cho et al. 2013; Kimura et al. 2013). Nevertheless, this study is the first, to the best of our knowledge, to investigate the impact of specific SNPs within the genes encoding these proteins on susceptibility and/or the severity of flavivirus infections. Further research is required to explore the role of these SNPs in patients infected with other flaviviruses.

In addition, further validation studies in larger TBE cohorts from different populations are needed to verify our findings. Genome-wide association studies (GWAS) may also be useful to determine the involvement of these and other genes in the development of TBE. In any case, this study provides new insights into our understanding of TBE pathogenesis and TBEV-host interactions.

## Data Availability

The data supporting the results of this study are available from the corresponding author upon reasonable request.
